# Mitigation of radiation-induced hematopoietic injury by the polyphenolic acetate 7, 8-diacetoxy-4-methylthiocoumarin in mice

**DOI:** 10.1038/srep37305

**Published:** 2016-11-16

**Authors:** Kavya Venkateswaran, Anju Shrivastava, Paban K. Agrawala, Ashok Prasad, Namita Kalra, Parvat R. Pandey, Kailash Manda, Hanumantharao G. Raj, Virinder S. Parmar, Bilikere S. Dwarakanath

**Affiliations:** 1 Division of Metabolic Cell Signalling Research, Institute of Nuclear Medicine and Allied Sciences, Brig. S. K. Mazumdar Marg, Lucknow Road, Delhi 110054, India; 2 Department of Zoology, University of Delhi, Delhi 110007, India; 3 Bioorganic Laboratory, Department of Chemistry, University of Delhi, Delhi 110007, India; 4 Department of Biochemistry, VP Chest Institute, University of Delhi, Delhi 110007, India; 5 Central Research Facility, Sri Ramachandra University, Porur, Chennai 600116, India

## Abstract

Protection of the hematopoietic system from radiation damage, and/or mitigation of hematopoietic injury are the two major strategies for developing medical countermeasure agents (MCM) to combat radiation-induced lethality. In the present study, we investigated the potential of 7, 8-diacetoxy-4-methylthiocoumarin (DAMTC) to ameliorate radiation-induced hematopoietic damage and the associated mortality following total body irradiation (TBI) in C57BL/6 mice. Administration of DAMTC 24 hours post TBI alleviated TBI-induced myelo-suppression and pancytopenia, by augmenting lymphocytes and WBCs in the peripheral blood of mice, while bone marrow (BM) cellularity was restored through enhanced proliferation of the stem cells. It stimulated multi-lineage expansion and differentiation of myeloid progenitors in the BM and induced proliferation of splenic progenitors thereby, facilitating hematopoietic re-population. DAMTC reduced the radiation-induced apoptotic and mitotic death in the hematopoietic compartment. Recruitment of pro-inflammatory M1 macrophages in spleen contributed to the immune-protection linked to the mitigation of hematopoietic injury. Recovery of the hematopoietic compartment correlated well with mitigation of mortality at a lethal dose of 9 Gy, leading to 80% animal survival. Present study establishes the potential of DAMTC to mitigate radiation-induced injury to the hematopoietic system by stimulating the re-population of stem cells from multiple lineages.

Injury to the hematopoietic system is a major contributing factor for the acute radiation effects in humans and other mammalian systems. The acute radiation syndrome (ARS) is characterized by a series of complex physiological and morphological developments manifesting eventually in multi-organ failure (MOF) resulting in the death of the organism^1^,^2^,^3^,^4^,^5^,^6^,^7^. The hematopoietic sub-syndrome of ARS (H-ARS) arises mainly due to the profound magnitude of damage to the actively proliferating cells in the hematopoietic system^8^,^9^,^10^,^11^ resulting in pancytopenia (anaemia, thrombocytopenia, neutropenia) owing majorly to the greater radio-sensitivity of the progenitor cells committed in these lineages^12^,^13^,^14^,^15^,^16^. The hallmarks of H-ARS include depletion of peripheral blood lymphocytes and consequently immunosuppression^12^,^17^ thereby, enhancing the susceptibility to opportunistic secondary infections^12^,^17^,^18^. Bone marrow (BM) is the most radio-sensitive tissue and transient myelosuppression involves loss of BM cellularity and damage to hematopoietic progenitor cells (HPCs) at moderate doses. However, at high doses (>3.5 Gy), BM failure occurs owing to severe injury to hematopoietic stem cells (HSCs) that can transform into long-term BM damage on complete ablation of HSCs reserves and functions^6^,^14^,^19^,^20^,^21^,^22^,^23^. Therefore, recovery and survival following exposure to myelo-ablative doses of TBI is majorly dependent on the maintenance of HSC reserves, their stem-ness and self-renewal capacity in addition to their ability to stimulate requisite levels of immune-competence^1^,^6^,^10^.

Shielding of spleen, transplantation of splenocytes, intravenous infusion of BM cell suspensions have been shown to enhance the survival of mice exposed to ionising radiation (IR) due to the presence of a “factor”, which has been identified as HSCs^24^,^25^,^26^,^27^ possessing self-renewal potential, and generating various progeny lineages in lethally irradiated mice following transplantation^27^,^28^,^29^. H-ARS in the absence of recovery can impose debilitating effects resulting in death within 4 weeks^12^,^30^,^31^ underscoring the importance of hematopoietic reconstitution as a chief criterion in development of medical countermeasures (MCMs) against IR.

A combination of cell death (apoptosis), cell proliferation and differentiation regulate the size of hematopoietic lineages such that imbalance in apoptosis of hematopoietic cells causes pathological conditions^6^,^32^. Following exposure to TBI, hematopoietic injury may be primarily due to the onset of apoptosis in BM cells and BM HSCs^20^,^33^. Autografts from mobilised blood stem cells to provide cell support after exposure to TBI as an intensive therapy for myelosuppression have been developed recently, but have several limitations^10^. Although, many strategies have been designed to counteract the H-ARS, they have shown limited efficacy and thus far none have been approved for human application by the FDA^12^. Therefore, there is a compelling need for developing countermeasure agents to ameliorate IR-induced hematopoietic injury. Acetylation is one of the vital post-translational modifications (PTMs), which regulates widespread functions in cells including chromatin-remodelling and gene expression^34^. Lysine acetylation orchestrates diverse cellular events in hematopoiesis by virtue of the lysine acetyl transferases (KATs/LATs) exerting histone/non-histone and catalytic/non-catalytic effects on the hematopoietic cells^35^. KATs such as p300 and CBP exercise pivotal control over hematopoietic stem and progenitor cells (HSPCs) by regulating processes such as self-renewal and differentiation^36^. Accumulating evidences suggest that KATs have critical functions in regulation of myeloid progenitors and their differentiation^35^ and mice homozygous for mutations in KIX domain of p300 exhibited several hematopoietic defects including B-cell deficiency, thymic hypoplasia, megakaryocytosis, anemia and thrombocytosis^37^. Furthermore, HSC-fate decisions are influenced by epigenetic events such that, chromatin-modifying agents expanded HSCs capable of marrow repopulation in CD34^+^ culture^38^. KDACi such as valproic acid (VA) prevent HSC loss by expansion of CD34^+^CD90^+^ cells, a function associated with increased histone acetylation^38^. *Ex vivo* expansion of human HSCs is induced by KDACi by potentiating engraftment in transplant patients in addition to HSC self-renewal by KDACi^39^.

Naturally occurring heterocyclic compounds such as the polyphenols have widespread biological and pharmacological implications^40^,^41^. Semi-synthetic acetyl derivatives of polyphenols such as 7, 8-diacetoxy-4-methylcoumarin (DAMC) and 7-acetoxy-4-methylcoumarin (7-AMC) have been shown to participate in protein acetylation of target proteins and alter their activity by virtue of novel acetoxy drug: calreticulin transacetylase (CRTase) acetylation system^42^,^43^,^44^,^45^,^46^,^47^ in addition to the therapeutic benefits of the parent moiety. DAMC, a polyphenolic acetate (PA) has been shown to acetylate target enzymes such as NADPH-cyto-c reductase, nitric oxide synthase (NOS), glutathione S transferase, cyto-P-450 in a calreticulin dependent manner and modulate their physiological activity^48^,^49^. DAMC up-regulates the expression and activity of anti-oxidant protein thioredoxin (TRX) in the peripheral blood mononuclear cells (PBMCs), accompanied by profound reduction in the levels of intracellular reactive oxygen species (ROS) levels thereby, minimising oxidative stress^50^. Accumulating evidences suggest that DAMC and other PAs lower Aflatoxin B_1_ (a potent carcinogen)-mediated micronuclei induction and afford protection against stress-induced DNA damage in normal lung and BM^51^. The potential of DAMC in stimulating angiogenesis by augmenting the levels of vascular endothelial growth factor (VEGF) has been recently demonstrated^50^. The novel protein acetylation system involving PAs and CRTase which mediates acetylation by virtue of acetyl group donating ability of the acetylated polyphenols, extends the domain of protein acetylation beyond the KATs as the PAs can potentially simulate the effects of acetylation produced by KATs. The molecule 7, 8-diacetoxy-4-methylthiocoumarin (DAMTC), being a PA with a sulphydryl group, possesses the potential to reduce oxidative stress, and thus, modify cellular responses to IR. By its ability to donate the acetyl group, DAMTC can also modify the dynamics of protein acetylation system, thereby, altering the cell signaling and epigenetic regulation through histone acetylation, extending its effects on the HSPCs maintenance, differentiation and self-renewal. Owing to the aforementioned, DAMTC can greatly influence the amelioration of hematopoietic injury and the H-ARS. The current study was aimed at evaluating the radio-protective and radio-mitigative efficacy of DAMTC against IR-induced hematopoietic injury and its implications on H-ARS. Therefore, we investigated the effects of DAMTC on IR-induced hematopoietic injury in C57BL/6 mice when administered 24 hours following lethal (9 Gy) and sub-lethal (7.6 Gy and 3 Gy) doses of TBI. Results clearly demonstrate that DAMTC ameliorates the radiation-induced damage to the hematopoietic system (H-ARS) thereby, facilitating recovery leading to mitigation of radiation-induced lethality as visualised by enhanced animal survival. Expansion of hematopoietic progenitor cells and reversal of BM suppression leading to the restoration of blood cell indices, coupled with immune modulation and induction of pro-inflammatory macrophages by DAMTC, appear to contribute to the mitigation of TBI-induced hematopoietic injury and the associated mortality.

## Methods

### Chemicals

7, 8-diacetoxy-4-methylthiocoumarin (DAMTC), whose synthesis has been reported earlier^52^ was obtained from the Department of Chemistry, University of Delhi, Delhi, India. Dimethyl sulfoxide (DMSO), Geimsa, Propidium iodide (PI), Ribonuclease-A (RNase-A) were obtained from Sigma-Aldrich Chemical Co., St. Louis, MO, USA. Methocult GF M3434 culture medium was purchased from Stem cell technologies, Canada. Antibodies to mouse F4/80 (FITC), MHC-II (PE), CD11b (APC) and CD86 (PerCP Cy5.5) were procured from eBiosciences, USA. Phosphate buffer saline (PBS), Ethylene diaminetetracetic acid (EDTA) and all other chemicals obtained were of analytical grade from BDH, Glaxo laboratories (Qualigens), SRL and E-Merck, India.

### Mice

Inbred C57BL/6 mice were obtained from the Institute’s central experimental animal facility. Female mice aged 8–10 weeks and weighing 20–25 grams were used in the study. Mice cages were housed in the facility maintained at 23–25 °C, relative humidity of 55 ± 5% with a 12-hour light/12-hour dark cycle and were fed standard rodent feed (from Golden Feeds, Delhi, India) and water *ad libitum*.

### Ethics statement

Animal studies were conducted in accordance with the policies of, and with the approval from, the Committee on the Ethics of Animal Experiments, Institute of Nuclear Medicine and Allied Sciences (INMAS), Defence Research and Development Organization (DRDO) (Institutional Ethical committee number under which this study has been approved is INM/IAEC/2011/08/001). All efforts were made to minimize suffering during the sacrifice of animals and experiments were carried out using protocols approved by the Committee on the Ethics of Animal Experiments of INMAS, DRDO.

### Irradiation

Mice were subjected to total body irradiation (TBI) in ^60^Co Gamma Teletherapy unit (Bhabatron II, Panacea Medical Technologies, Bangalore, India) at a dose rate of 1 Gy/min. (dose rate of the ^60^Co Gamma irradiation source was calibrated using physical dosimetry). All irradiations were carried out at room temperature. For the radio-protection/mitigation studies mice were subjected to a lethal dose of 9 Gy. Evaluation of effects of DAMTC on TBI-induced H-ARS was performed by exposing mice to sub-lethal doses of 7.6 Gy and 3 Gy.

### Preparation and administration of 7, 8-diacetoxy-4-methylthiocoumarin (DAMTC)

DAMTC was prepared fresh each time by dissolving in dimethyl sulfoxide (DMSO) and diluting in water to a final concentration of 0.1% DMSO and administered by intra-peritoneal injection of 5 μg/kg. DAMTC was administered one and four hours prior to TBI for radio-protection studies. For the radio-mitigation studies, DAMTC was injected 24 hours following TBI. Subsequent experiments to elucidate the mechanism underlying radio-mitigation involving effects on the hematopoietic system were all carried out by administering DAMTC at 24 hours post TBI.

### Survival studies and evaluation of dose-modifying factor (DMF)

Mice were administered DAMTC prior to (one and four hours) and post (24 hours) TBI (9 Gy) and monitored for survival through 30 days. To calculate the DMF of DAMTC, mice were subjected to sub-lethal (5 Gy, 7.6 Gy), lethal (9 Gy) and supra-lethal (9.5 Gy, 10 Gy, 10.5 Gy and 11 Gy) doses of TBI and monitored for survival through 30 days. The DMF of DAMTC at LD_50/30_ was calculated as:


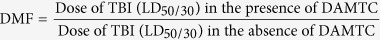


### Peripheral blood analysis

Blood was directly withdrawn from the retro-orbital plexus and collected in pre-coded EDTA containing vials on days 3, 7, 14, 21 and 28 following treatment. Blood was mixed gently on a rotary shaker until analysis of red blood cells, haemoglobin, platelets, white blood cells, and lymphocytes on a hematology analyser (MEK-6400; Nihon Kohden, Japan) and data was generated using DMS-Lite software (software provided with the hematology analyser).

### Endogenous colony-forming units in spleen (e-CFU-S)

The classical e-CFU-S was performed essentially as described earlier^53^. Briefly, mice were euthanized on day 10 following the treatment and spleens were isolated. Spleens were then fixed in Bouin’s solution (SRL) and examined with naked eye for macroscopic colonies on the splenic surface.

### Histological examination of bone marrow

Mice were euthanized and femurs isolated on day 10 post treatment. Femurs were then fixed in 10% neutral-buffered formalin (SRL) followed by decalcification by immersing the femurs in 10% EDTA (pH 7–7.4) at 4 °C for 4–5 days. Femurs were then embedded in paraffin for micro-section into 5 μm slices, placed on slides and stained with hematoxylin and eosin (H&E). The slides were examined by light microscopy to capture bright-field images using Olympus (IX51) microscope (Japan).

### Bone marrow cell harvesting and cell counting

Femurs were isolated post euthanasia of mice on days 10 and 45 following the treatment. Bone marrow cells were flushed from isolated femurs using 26-gauge needle with pre-chilled phosphate-buffered saline (PBS). Single cell suspension was prepared by gentle pipetting of the flushed cells few times and number of viable bone marrow nucleated cells (BMNCs) was counted using a hemocytometer (Neubauer, Marienfeld, Germany) and expressed as ×10^6^/femur.

### *Ex vivo* hematopoietic CFC assay (colony forming cells assay) in the bone marrow

Mice were sacrificed under aseptic conditions and femurs were isolated on day 10 post treatment. Bone marrow cells were flushed from the isolated femurs (as described above) with PBS supplemented with 2% fetal bovine serum after removing excess tissue from the femurs and cutting the epiphyseal ends. Single-cell suspension was prepared and allowed to pass through 100 μm nylon mesh strainer to remove debris and clumps. Cells were washed twice with PBS and counted using a hemocytometer. Cells were plated *ex vivo* at a concentration of 5 × 10^4^ cells per 35 mm cell culture dish in Methocult GF M3434 (Stemcell Technologies, Canada) growth medium according to the manufacturer’s protocol and incubated for 12–14 days at 37 °C, 5% CO_2_, 95% humidity. Colonies were visualised on day 12 post culture using a light microscope (Olympus IX51, Japan) and scanned in a 60 mm gridded scoring Petri dish (Stemcell Technologies, Canada) to identify and enumerate hematopoietic CFCs namely CFU-GM (CFU-granulocyte, macrophage), CFU-GEMM (CFU- granulocyte, erythroid, macrophage, megakaryocyte) and BFU-E (Burst-forming unit-erythroid). Data is reported as different CFUs as well as cumulative CFUs from individual mouse.

### Bone marrow micronucleus assay

Mice were sacrificed at 24, 48 and 72 hours after treatment and femurs were isolated followed by flushing of bone marrow cells with pre-chilled PBS as described above. The cell suspension was processed for the micronucleus assay essentially according to Schmid’s method^54^ with minor modifications. Briefly, cells were washed in PBS, re-suspended in few drops of FBS and smeared on a clean dry glass slide. The slides were air-dried, fixed in absolute methanol and stained with May-Grünwald Geimsa stain (1:1) in Sorensen’s phosphate buffer (pH 6.8) for 20–30 minutes, followed by washing to remove excess stain. The slides were examined for micronucleated polychromatic erythrocytes (MN-PCEs) by scoring a minimum of 1000 PCEs under light microscope by an independent person and data is represented as frequency of MN-PCEs per 1000 PCEs. The slides were decoded on completion of scoring.

### Peripheral blood micronucleus assay

Blood was withdrawn from the retro-orbital plexus of mice and collected in EDTA containing vials at 24, 48 and 72 hours following treatment. Blood collected was smeared on clean, dry glass slides, air-dried, fixed and stained with May-Grünwald Geimsa stain as described above. The blood smears were examined for MN-PCEs by scoring a minimum of 2000 erythrocytes under light microscope by an independent person and data is represented as frequency of MN-PCEs per 2000 erythrocytes. The slides were decoded on completion of scoring. Blood smears were prepared as described above and stained with acridine orange (AO) to visualise erythrocytes^55^. Briefly, blood smears were stained with AO (125 μg/ml in pH 6.8 phosphate buffer) for two minutes and visualised under Olympus (IX51) microscope (Japan) to capture fluorescence images. The AO-stained blood smears were examined for PCEs and MN-PCEs in a total of 2000 erythrocytes.

### Analysis of apoptotic cell death and proliferation status in the bone marrow

Mice were euthanized at days 3, 21 post treatment and 24, 48 and 72 hours post treatment when exposed to 7.6 Gy and 3 Gy respectively. Femurs were isolated and bone marrow cells collected as described above. The cells were fixed in 80% chilled ethanol and cell cycle distribution was studied to assess the hypo-diploid peak (indicator of apoptotic cell death) and S-phase cells (index of proliferating cells). Briefly, about 0.5 million fixed cells were washed in PBS, treated with Ribonuclease-A (200 μg/ml) for 30 minutes at 37 °C and subsequently stained with Propidium Iodide (50 μg/ml) in PBS for 15 minutes at 4 °C. Measurements were made with a laser based (488 nm) flow cytometer (FACS Calibur; Becton and Dickinson, USA) and data acquired using the Cell Quest software (Becton and Dickinson, USA). Cell cycle analysis was performed using the Mod Fit program (Becton and Dickinson, USA).

### Isolation of splenocytes and characterization of macrophages

Mice were sacrificed at 24, 48, 72 hours post treatment and spleens were isolated under aseptic conditions. Single-cell suspension from spleen was prepared by placing in between two frosted slides and gentle crushing and mincing in pre-chilled PBS, followed by gentle pipetting few times and passed through 70 μm nylon mesh to remove debris and washed in PBS. After RBC lysis, splenocytes were washed twice with PBS. About 2 million cells were re-suspended in 100 μl flow staining buffer along with the fluorochrome-conjugated antibodies against F4/80 (FITC), MHCII (PE), CD11b (APC) and CD86 (PerCP Cy5.5) and incubated for 1 hour. After the incubation, cells were again washed in PBS to remove unbound antibodies and re-suspended in 500 μl flow staining buffer. Samples were then acquired on BD LSR II and analyzed using FACS Diva software.

### Statistical analysis

All the data were analyzed using Graph Pad Prism (version 5.01) and is presented as mean ± standard error of mean (SEM). Significance of difference between groups was determined by Student’s t test and One-way or two-way ANOVA with Tukey’s, Newman-Keuls’, Dunnett’s or Bonferroni’s multiple comparison post-tests. Survival studies’ data were analysed using the Kaplan-Meier method followed by Mantel-Cox (log-rank) and Gehan-Breslow-Wilcoxon tests for assessment of significant differences. Results were considered significant at P < 0.05.

## Results

### *In vivo* radio-mitigation by DAMTC

**Figure 1:**
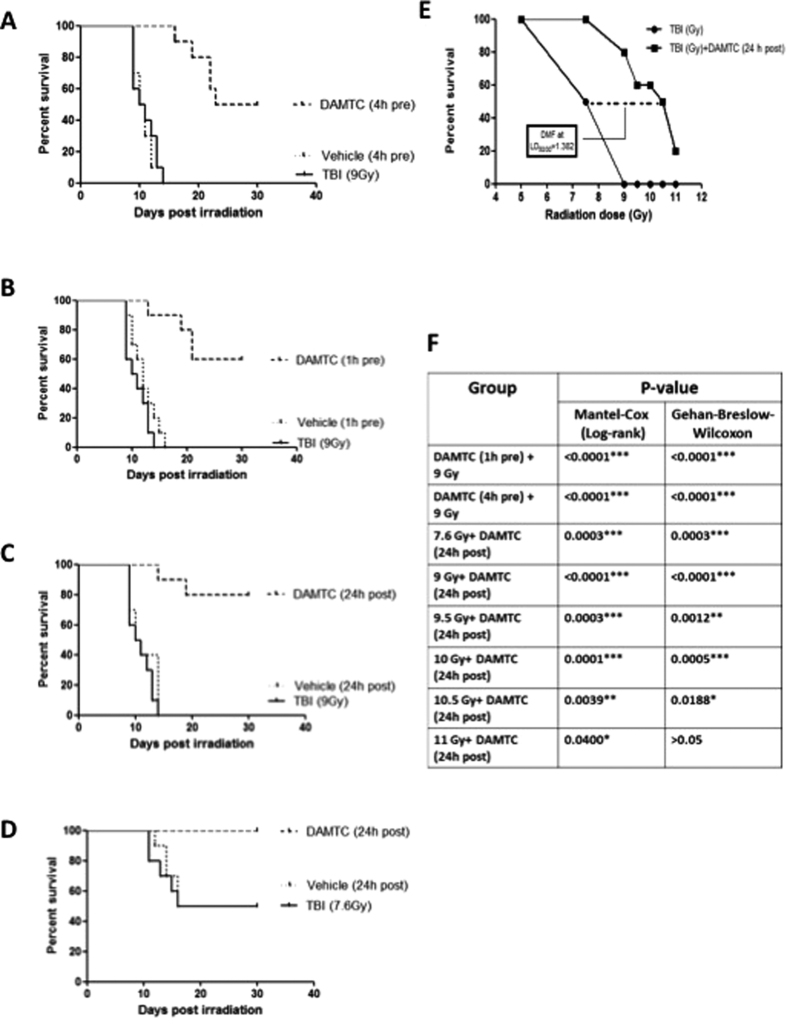
DAMTC mitigates TBI-induced lethality in mice. (**A**,**B**) Protective effects of DAMTC (i.e. administered prior to irradiation) against lethal TBI (9 Gy). Mice were injected DAMTC intra-peritoneally and irradiated 1 or 4 hours later. Kaplan-Meier survival curve depicts the 30 day survival (n = 20 in all the groups i.e. TBI, Vehicle + TBI, DAMTC + TBI). (**C**,**D**) Mitigative effects of DAMTC administered 24 hours post TBI is represented in the Kaplan-Meier survival curves, when mice were exposed to lethal TBI (9 Gy; (**C**)) and sub-lethal TBI (7.6 Gy; (**D**)). In both (**C**) and (**D**) n = 20 for all the groups (TBI, TBI + Vehicle, TBI + DAMTC). (**E**) Dose response of TBI on mice and DMF evaluation of DAMTC at LD_50/30_. Mice were subjected to varying TBI doses (5–11 Gy) and treated with DAMTC or vehicle at 24 hours following irradiation and monitored for survival through 30 days (n = 10). (**F**) Table summarises the findings of Mantel-Cox (log-rank) and Gehan-Breslow-Wilcoxon statistics for comparing survival responses between TBI and TBI + DAMTC in experiments used for generating the DMF shown in (**E**). *P < 0.05; **P < 0.01; ***P < 0.001.

DAMTC administered prior to irradiation conferred significant survival benefit, which was dependent on the time gap between irradiation and administration of DAMTC. At the lethal radiation dose (9 Gy and 0% survival), a 50% animal survival was observed at day 30 post TBI, when DAMTC was administered 4 hour prior to irradiation (p < 0.0001) (Fig. 1A) and 60% survival (p < 0.0001; Fig. 1B) when administered 1 h prior to irradiation. At this radiation dose, the entire cohort of animals died on (or before) 15–16 days post TBI accompanied with symptoms of radiation sickness such as weight loss, diarrhoea, facial oedema, anorexia, lethargy and delacrymation. To assess the radio-mitigative effects of DAMTC, mice were subjected to lethal TBI (9 Gy) and DAMTC was administered 24 hours post TBI. The results suggesting 80% survival in the DAMTC-TBI mice (p < 0.0001) (Fig. 1C) at day 30 post TBI is noteworthy, which was even higher than the survival seen when DAMTC was administered prior to TBI (i.e. the protective effects). The significant radio-mitigation by DAMTC at 24 hours post lethal TBI of 9 Gy prompted us to ascertain the dose modifying factor (DMF) of DAMTC. To calculate the DMF for mitigation, mice were exposed to varying doses of TBI at sub-lethal (5 Gy and 7.6 Gy), lethal (9 Gy) and supra-lethal (9.5 Gy, 10 Gy, 10.5 Gy and 11 Gy) doses followed by DAMTC administration 24 hours post TBI. The 30 day survival was 100% in the DAMTC group as against 50% in animals receiving only the vehicle at 7.6 Gy TBI (Fig. 1D). Notably, at all supra-lethal doses of TBI, DAMTC exhibited significant mitigation as evident from the animal survival of 60% at 9.5 Gy (p = 0.0003), 60% at 10 Gy (p = 0.0001), 50% at 10.5 Gy (p = 0.0039) and 20% at 11 Gy (p = 0.04) on day 30 post TBI as compared with radiation alone group experiencing absolute mortality (Fig. 1E,F). The DMF of DAMTC at LD_50/30_ was calculated as 1.382 (Fig. 1E). Subsequent studies to unravel the contributing factors for mitigation of radiation-induced injury to the hematopoietic system by DAMTC (H-ARS) were carried out at the LD_50/30_dose of 7.6 Gy.

### Mitigation of H-ARS by DAMTC following TBI: Facilitation of hematopoietic recovery following TBI

#### Alterations in hematological indices in the peripheral blood

**Figure 2:**
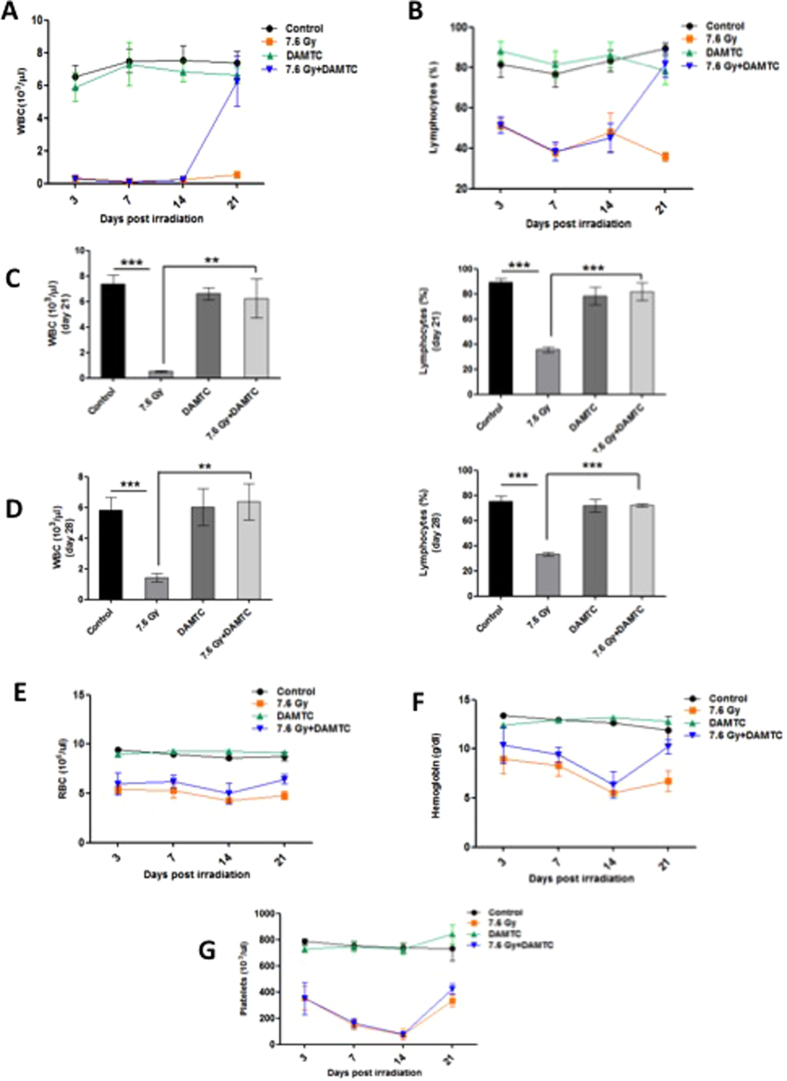
DAMTC mitigates TBI-induced pancytopenia in mice. Alterations in the hematological indices in the peripheral blood by DAMTC. Mice were subjected to TBI (7.6 Gy) followed by the administration of DAMTC at 24 hours and assessed for blood cell parameters. Blood counts showing (**A**) WBC (**B**) Lymphocyte at days 3, 7, 14, 21 post TBI. (**C**,**D**) WBC, lymphocyte levels showing the effects of DAMTC observed on day 21 (**C**) and day 28 (**D**). Blood counts showing (**E**) RBC, (**F**) hemoglobin, (**G**) platelet at days 3, 7, 14, 21 post TBI (n = 10). All error bars indicate SEM. *P < 0.05; **P < 0.01; ***P < 0.001.

Damage to hematopoietic system (H-ARS) mainly contributes to the acute radiation effects, with mortality as one of the end points^6^,^12^. Therefore, we investigated the effects of DAMTC on radiation-induced pancytopenia and myelosuppression by exposing mice to 7.6 Gy followed by DAMTC administration 24 hours later. Assessment of the peripheral blood for various hematological parameters collected from the animals at days 3, 7, 14 and 21 post TBI revealed reduction in all the constituent parameters following irradiation (Fig. 2). WBC counts in the irradiated animals decreased significantly at day 3 (p < 0.001), compared to the naïve (un-irradiated control/sham-irradiated) animals. There was no improvement in the WBC levels through day 21 in the TBI mice (p < 0.001). On the other hand, DAMTC modulated the WBC count by reversing the blunted WBC nadir by a rapid and drastic recovery in the WBC counts leading to the attainment of the baseline levels by day 21 (p > 0.05 compared to naïve mice) (Fig. 2A), which was nearly 12 folds higher (p < 0.0004) than the levels seen in the TBI cohort (Fig. 2C), suggesting a profound acceleration in the recovery of the WBC index in the peripheral blood of irradiated animals by DAMTC. The percentage of lymphocytes in the TBI cohort showed drastic fluctuations with a significant reduction at day 3 (p < 0.01), reaching a nadir at day 7 (p < 0.001), followed by a marginal recovery at day 14, which was still significantly lower than the naïve mice (p < 0.001). The reduction in the percentage of lymphocytes by TBI was significantly reversed by DAMTC reaching the baseline values at day 21 (Fig. 2B). More than 2 folds higher level of lymphocytes observed (p = 0.0004) is indicative of an accelerated recovery mediated by DAMTC following TBI (Fig. 2C). We also examined the status of lymphocytes and WBCs at day 28 post TBI and found that complete recovery is not achieved in the TBI mice (1.44 × 10^3^ ± 0.284 cells μl^−1^; p = 0.0049), while a complete recovery is facilitated by DAMTC (6.367 × 10^3^ ± 1.18 cells μl^−1^Vs 5.84 × 10^3^ ± .0809 cells μl^−1^; p = 0.0009) (Fig. 2D). The significantly higher fold change of WBCs over radiation alone group (3.6 folds; p = 0.0018) observed with DAMTC treatment at day 28 further corroborated the accelerated and sustained hematopoietic recovery facilitated by DAMTC post TBI (Fig. 2D). Percentage of lymphocytes remained significantly lower in the TBI mice (33.43 ± 1.5%; p < 0.0001) at day 28 compared to naïve mice (75.32 ± 3.8%), while DAMTC treatment brought lymphocyte levels comparable to the naïve cohort (72.2 ± 1.3%; p < 0.0001) (Fig. 2D). The levels of RBC were lowered by day 3 (p < 0.001) reaching its minimum at day 14 (p < 0.001), and the recovery was not achieved even at day 21 (p < 0.001) (Fig. 2E). DAMTC treatment mediated partial recovery in RBC counts of irradiated animals at day 21 compared to the radiation alone cohort, which was not significant. The levels of hemoglobin dropped at day 3 (p < 0.01), day 14 (p < 0.001) and remained low compared to the naïve animals at day 21 (p < 0.01). On DAMTC treatment, a substantial recovery, almost reaching the baseline values at day 21 was observed (Fig. 2F). The platelet levels exhibited a similar pattern as RBC displaying a reduction at day 3 (p < 0.001), day 14 (p < 0.001) and failing to achieve recovery at day 21 (p < 0.001). DAMTC minimally altered the platelet levels in the irradiated animals with a marginal, but insignificant recovery at day 21 (Fig. 2G). Taken together, these results reveal the potential of DAMTC in reversing radiation-induced leukopenia in mitigating H-ARS and TBI-induced lethality in C57BL/6 mice.

#### Restoration of hematopoietic potency in splenic progenitor cells

**Figure 3:**
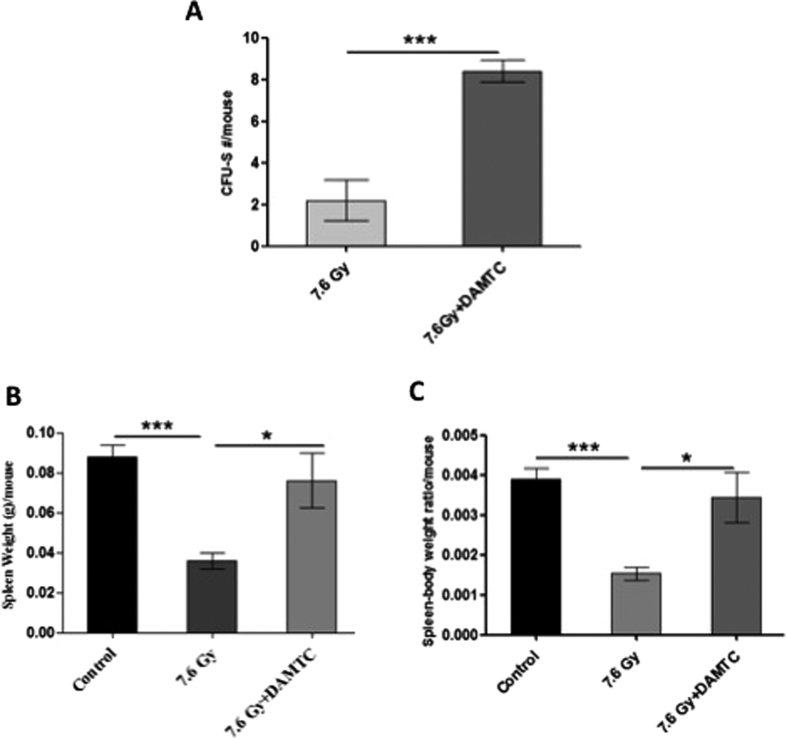
DAMTC mediates stimulation of the splenic progenitor cells to mitigate TBI-induced hematopoietic damage in mice. Mice were subjected to TBI (7.6 Gy) followed by the administration of DAMTC at 24 hours and assessed for endogenous splenic colonies on day 10. (**A**) Spleen was fixed in Bouin’s solution to enumerate the CFUs. Representation of CFU-S in TBI (n = 12) and DAMTC + TBI (n = 12). (**B**) Spleen weight and (**C**) spleen-body weight ratio (n = 12). All error bars indicate SEM. *P < 0.05; **P < 0.01; ***P < 0.001.

TBI-induced myelosuppression is visualised in every compartment of the hematopoietic system^6^,^12^. The numbers of endogenous CFUs serve as an indicator for hematopoiesis, a crucial and indispensable factor dictating hematopoietic recovery post TBI and consequently survival^10^,^17^. To determine the effects of DAMTC on hematopoiesis and recovery following TBI, precisely the splenic HSPCs, endogenous spleen colony forming assay was performed. The number of endogenous CFUs in TBI mice was 2.2 ± 0.97, which was increased by nearly 3.8 folds (8.4 ± 0.51; p = 0.0005) in animals that received DAMTC (Fig. 3A), suggesting that DAMTC significantly accentuated the number of splenic colonies in the irradiated animals. To assess the effect of DAMTC on TBI-induced splenic atrophy, spleen weight was examined at the end of 10 days of treatment. A significant diminution of splenic size was observed in the TBI mice, which on an average was ~2.5 folds lower compared to the size of the spleen in un-irradiated mice (p < 0.0001). On the other hand, splenic atrophy was not apparent in the TBI mice that received DAMTC and was comparable to the size (as well as weight) of the naïve mice (p > 0.05) (Fig. 3B). The spleen to body weight ratio was also found to be markedly reduced in the TBI mice (p = 0.0001), while it was comparable to naïve animals in the cohort administered DAMTC following TBI (p < 0.05) (Fig. 3C). These observations suggest that alleviation of the TBI-induced splenic atrophy and stimulation of endogenous CFU-S by DAMTC also contribute to the mitigation of hematopoietic injury.

#### Restoration of bone marrow cellularity and stimulation of hemopoiesis

**Figure 4:**
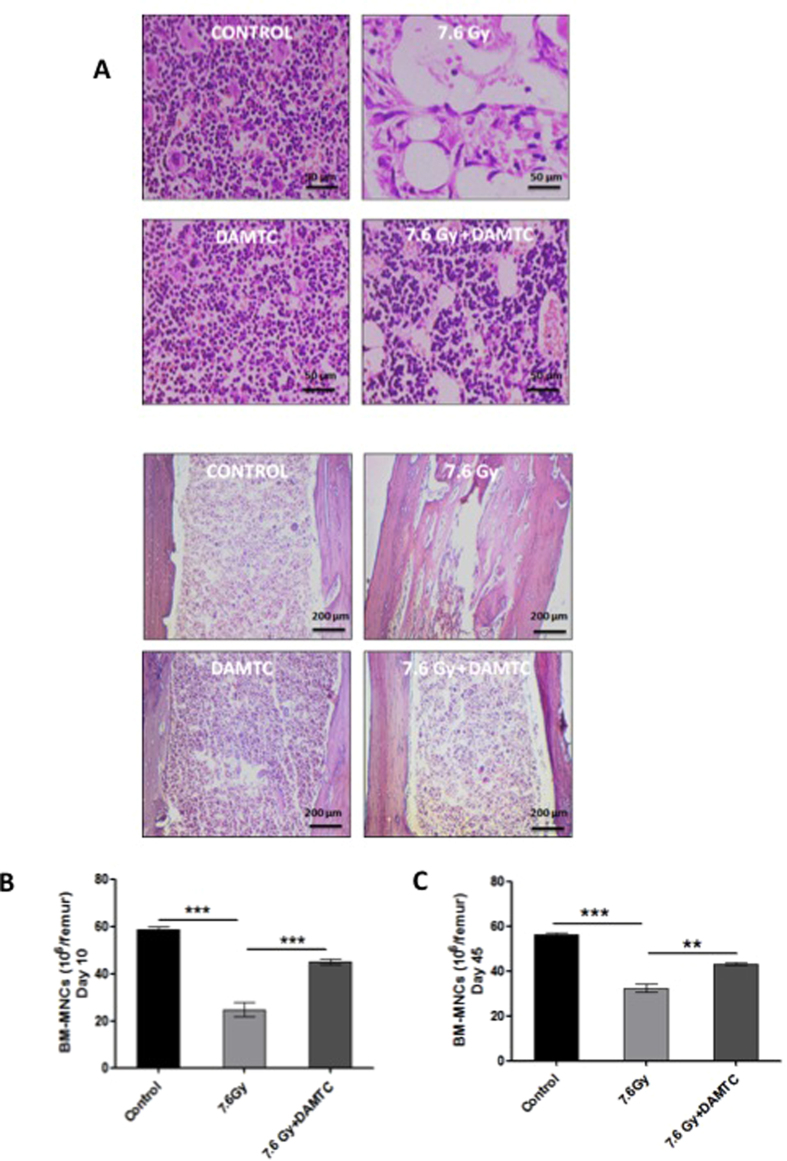
DAMTC mitigates TBI-induced BM failure in mice. Effects of DAMTC on bone marrow (BM) cellularity in TBI mice. (**A**) Panels show H&E staining of mouse femurs. Representative images are shown for naïve, DAMTC, TBI, and TBI + DAMTC treatments. For the upper panel, scale bar = 50 μm with an original magnification of ×400. For the lower panel, scale bar = 200 μm with an original magnification of ×100. (**B**) Total nucleated cell numbers of BM seen in naïve, TBI, TBI + DAMTC mice at day 10 and (**C**) day 45 post TBI (cells from ten animals were examined in each group; n = 10). All error bars indicate SEM. *P < 0.05; **P < 0.01; ***P < 0.001. Imaging of H&E stained femurs was done on day 10 post TBI.

TBI-induced BM suppression is a major contributing factor towards IR-induced hematopoietic injury and is characterized by loss of the BM cellularity^13^,^56^. Restoration of the bone marrow cellularity is suggestive of preservation and stimulation of the constituent stem cells^6^,^12^. The effects of DAMTC on the radiation-induced damage to the hematopoietic stem cells (HSCs) from the BM were analysed by histological examination of the femurs on day 10 post TBI (7.6 Gy). Severe radiation-induced disruption of BM HSCs and vasculature leading to massive ablation of cellular content of the BM was evident in TBI mice (Fig. 4A). In contrast, DAMTC administration reversed the radiation-induced cellular depletion to a large extent with cell numbers and cellular content almost similar to levels in the naive animals (Fig. 4A). This indicates that DAMTC mitigates the TBI-induced BM hypo-cellularity by enhancing hemopoiesis and facilitating stem cell regeneration to accelerate the hematopoietic recovery. We determined the number of bone marrow nucleated cells (BMNCs) at days 10 and 45 post TBI. At day 10, nearly 60% decrease was seen in the BMNC count in the TBI mice (24.86 × 10^6^ ± 3.11/femur; compared to 58.73 × 10^6^ ± 1.27/femur in the naive mice; p < 0.0001) (Fig. 4B). On the contrary, the BMNC count in the TBI mice treated with DAMTC (45.1 × 10^6^ ± 1.18/femur; p = 0.0003), was nearly 2 folds higher (p = 0.0002) (Fig. 4B). At day 45, the BMNC counts in TBI mice (32.33 × 10^6^ ± 1.79/femur; p < 0.0001) was still significantly lower than the levels in the naive mice (56.4 × 10^6^ ± 0.83/femur) (Fig. 4C), suggestive of incomplete recovery. Although the BMNC counts in mice that received DAMTC (43.23 × 10^6^ ± 0.45/femur; p < 0.05) (Fig. 4C) was higher compared to the radiation alone cohort, they were similar to the value observed at day 10, suggestive of saturation in the recovery by day 10 (Fig. 4C). Taken together, these results provide an evidence for the amelioration of TBI-induced loss of BM cellularity and proliferative capacity of the BM cells by DAMTC.

#### Expansion of hematopoietic progenitor cells in the bone marrow

**Figure 5:**
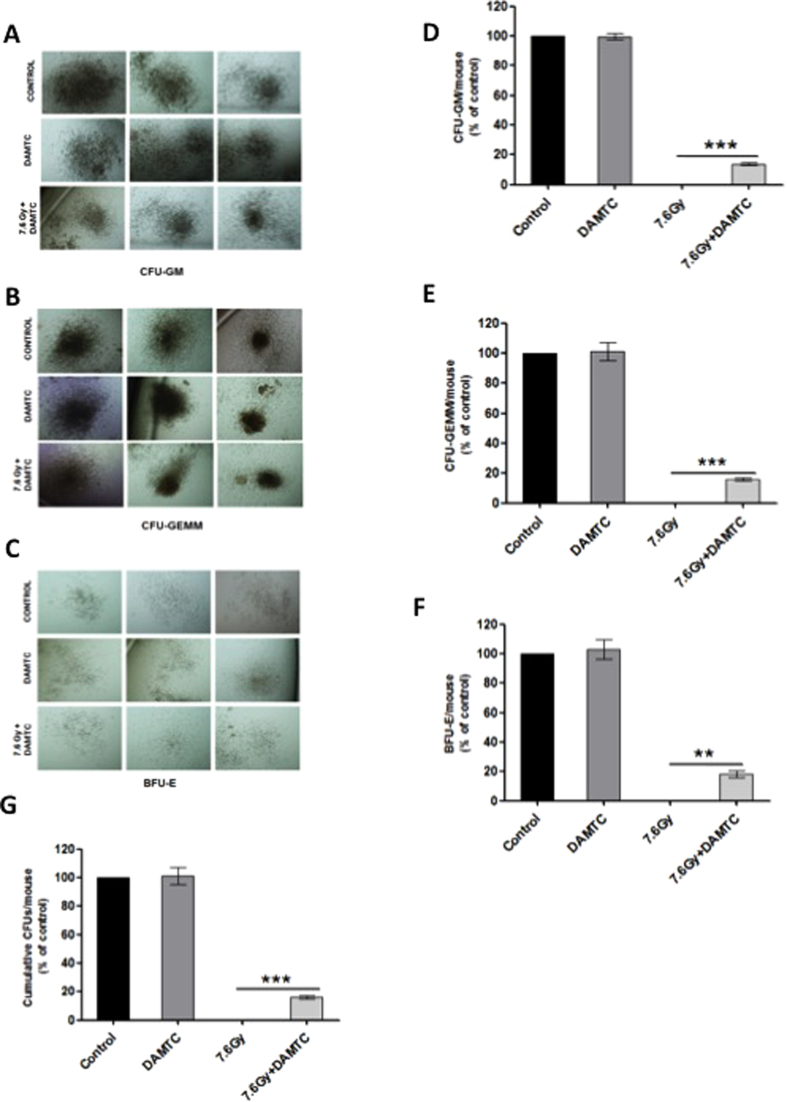
DAMTC facilitates expansion of hematopoietic progenitors in the BM of TBI mice. Effects of DAMTC on BM hematopoietic progenitor cells (HPCs) in TBI mice. Panels show colonies of hematopoietic progenitors (**A**) CFU-GM, (**B**) CFU-GEMM and (**C**) BFU-E after performing *ex-vivo* culturing on day 10 post TBI. Representative images of colonies from naïve, DAMTC and TBI + DAMTC mice are shown (cells from ten animals were examined in each group; n = 10). Percentages of (**D**) CFU-GM, (**E**) CFU-GEMM, (**F**) BFU-E and (**G**) cumulative CFUs are shown. All error bars indicate SEM. *P < 0.05; **P < 0.01; ***P < 0.001. Imaging of BM hematopoietic CFUs was done on day 12 of *ex-vivo* culture. Original magnification, ×40 (**A**–**C**).

To determine the effects of DAMTC on TBI-induced depletion of HPCs i.e. colony forming cells (CFCs) in the BM, and to understand whether DAMTC mediated hematopoiesis in the BM following TBI is due to the stimulation of hematopoietic progenitor cells (HPCs), we performed the *ex-vivo* hematopoietic CFC assay. The myeloid progenitors examined were burst forming unit-erythroid (BFU-E), colony forming unit-granulocyte, macrophage (CFU-GM) and colony forming unit-granulocyte, erythroid, macrophage, megakaryocyte (CFU-GEMM) (Fig. 5A,B,C). A complete abrogation of all the myeloid progenitors was evident in the TBI cohort (Fig. 5A,B,C). On DAMTC treatment, the irradiated mice displayed a dramatic upregulation of CFU-GM counts (18.33 ± 1.45; p < 0.0001) compared with the TBI mice (0.2 ± 0.13) (Fig. 5D) indicating about a 90 folds increase. The BFU-E counts in the DAMTC-treated cohort (16.67 ± 2.4; p < 0.05), indicated a nearly 50 fold increase in the numbers of the myeloid progenitor BFU-E when compared with the TBI mice (0.33 ± 0.15) (Fig. 5F). The myeloid progenitor CFU-GEMM was totally abolished in the TBI mice, whereas significant numbers of CFU-GEMM counts (3.333 ± 0.67, p < 0.05) were observed on DAMTC treatment post TBI (Fig. 5E). The enumeration of cumulative counts of all the myeloid progenitors was suggestive of an enormous ~75 folds higher numbers of the progenitors on DAMTC treatment in TBI mice (38 ± 3.05; p < 0.0001), in comparison with the radiation alone mice (0.5 ± 0.26) (Fig. 5G). Thus, reduction in the TBI-induced depletion of BM hematopoietic progenitor cells and restoration of functionality by DAMTC contributes to recovery from H-ARS.

#### Attrition of radiation-induced cytogenetic damage in the bone marrow

**Figure 6:**
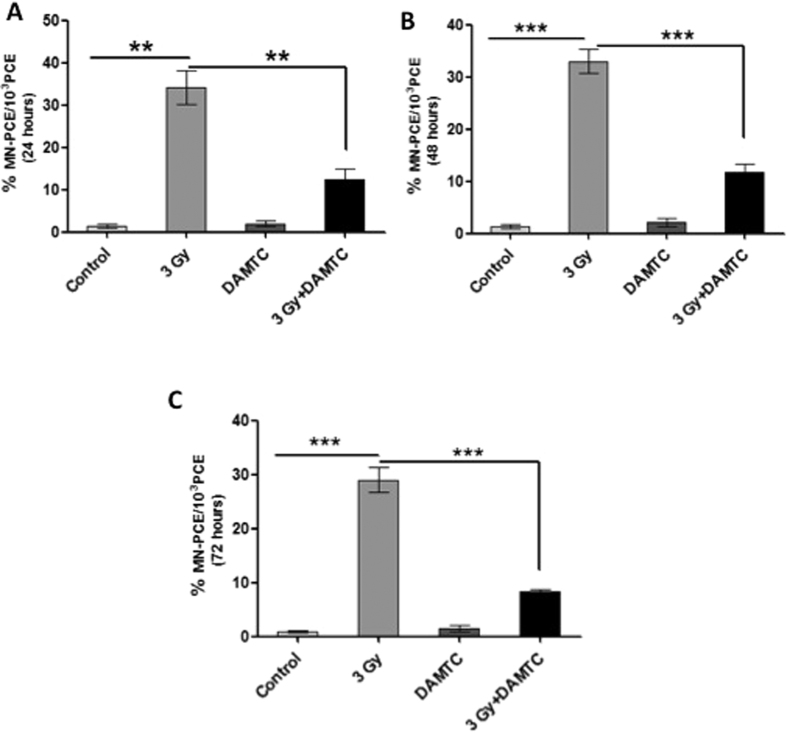
DAMTC mitigates TBI-induced cytogenetic damage in BM. Effects of DAMTC on TBI-mediated MN-PCEs in mouse BM. BM smears were evaluated for MN induction by May-Grϋnwald-Geimsa staining. Representation of frequency of MN-PCEs per 10^3^ PCEs at (**A**) 24 hours (**B**) 48 hours and (**C**) 72 hours following DAMTC treatment in naïve, DAMTC, TBI (3 Gy) and TBI + DAMTC mice (n = 10 in all groups). All error bars indicate SEM. *P < 0.05; **P < 0.01; ***P < 0.001.

Mitotic death contributes majorly towards TBI-induced lethality^2^ and in the different hematopoietic compartments, DNA damage is also responsible for TBI-induced hematopoietic injury. Radiation-induced cytogenetic damage (mitotic death) in the BM manifests as micronuclei in the polychromatic erythrocytes. During maturation, erythroblasts develop into a polychromatic erythrocyte and extrudation of the main nucleus occurs allowing for distinct visualisation of micronuclei in the polychromatic erythrocytes as an indicative measure of induced chromosomal damage^57^,^58^. To examine the potency of DAMTC in reducing the radiation induced cytogenetic damage in the hematopoietic compartment, as the same plays an essential role in myelosuppression, we performed the MN assay in the BM. Although, changes in the percentage micronuclei in the polychromatic erythrocytes is not sufficient for concluding the effects of any intervention on the induced cytogenetic damage in other cell populations of the BM, erythrocytes were chosen, as they have important implications in BM hematopoiesis and also since IR, is known to deplete the rapidly dividing cells of the BM^57^,^58^. The frequency of MN-PCE in the naïve cohort was found to be 1.47% ± 0.437. At 24 hours of treatment, drastic elevation in the MN frequency was visualised in the PCE of the TBI mice (34.23% ± 4.05; p < 0.01). DAMTC significantly reduced the radiation-induced MN frequency (12.35 ± 2.45; p < 0.01) (Fig. 6A). At 48 h, the MN frequency in the TBI cohort remained substantially higher (33% ± 2.317; p < 0.0001) than the naïve cohort (1.33% ± 0.405), which was significantly reduced (11.83% ± 1.56; p < 0.001) by DAMTC (Fig. 6B). A similar trend was noted at the end of 72 h following irradiation (Fig. 6C). These results provide an unequivocal evidence for the potential of DAMTC to attenuate the radiation-induced cytogenetic damage in the BM. These observations with DAMTC, are similar to Ex-RAD, a chlorobenzylsulfone derivative, which has been recently shown to mitigate radiation induced hematological injury when administered 24–36 hours following TBI exposure^59^.

#### Reduction in the radiation-induced cytogenetic damage in the peripheral blood

**Figure 7:**
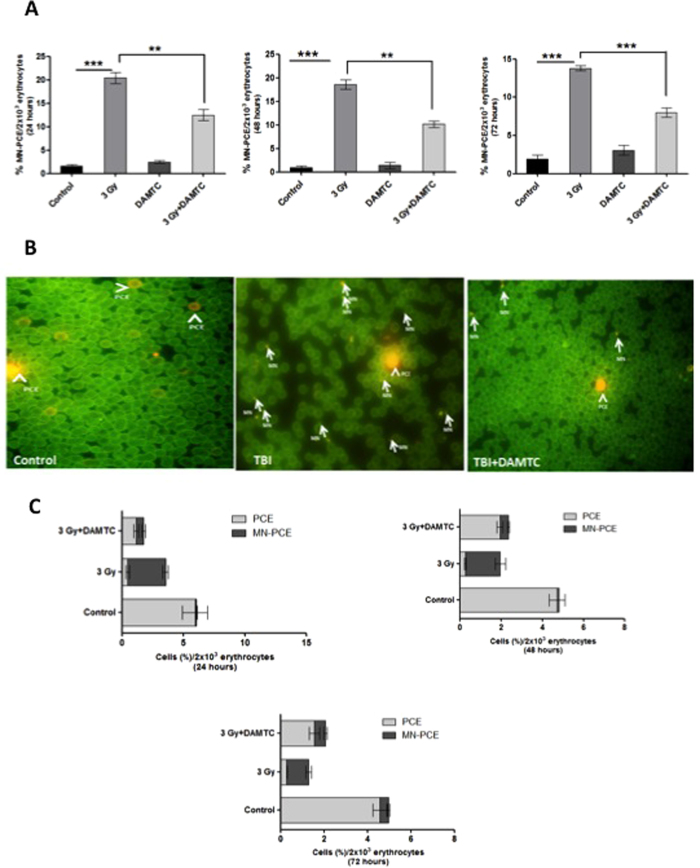
DAMTC mitigates TBI-induced cytogenetic damage in the peripheral blood of TBI mice. Effects of DAMTC on TBI-induced MN-PCEs in mouse blood. Blood smears were evaluated for MN induction by May-Grϋnwald-Geimsa staining. Representation of frequency of MN-PCEs per 2 × 10^3^ erythrocytes at (**A**) 24, 48 and 72 hours following DAMTC treatment in naïve, DAMTC, TBI (3 Gy) and TBI + DAMTC mice (n = 10 in all groups). Blood smears were stained with acridine orange (AO) to assess the effects of DAMTC on TBI-mediated cytotoxicity and genotoxicity. (**B**) Panels showing representative images of AO stained blood smears from naïve, TBI and TBI + DAMTC mice (Original magnification, ×400). Arrow heads and arrows represent PCE and MN-PCE respectively. (**C**) Frequency of PCEs and MN-PCEs per 2 × 10^3^ cells at 24, 48 and 72 hours visualised by AO staining. All error bars indicate SEM. *P < 0.05; **P < 0.01; ***P < 0.001.

Cells with micronuclei are generally associated with mitotic death^60^, eventually leading to hematopoietic toxicity. The MN-PCE frequency in the blood smears of TBI mice was highest at 24 hours of treatment (20.4% ± 1.21; p = 0.0001) followed by a decrease, reaching a value of 13.8% ± 0.33 (p < 0.0001) at 72 h, significantly higher than the basal value (0.96–1.9%) observed in the naive mice (Fig. 7A). Administration of DAMTC significantly reduced the radiation-induced MN frequency and was nearly 60% of the values seen in TBI mice at all the three observed time intervals (Fig. 7A). These results reiterated the efficacy of DAMTC in subjugation of radiation-induced cytogenetic damage, in the peripheral blood.

An alternate methodology based on acridine orange fluorescent staining for the assessment of the frequency of immature-erythrocytes i.e. polychromatic erythrocytes (PCEs) and MN-PCEs was also adopted to ascertain the effects of DAMTC on TBI-induced cytotoxicity and genotoxicity respectively in the peripheral blood. A total of 2000 cells were evaluated comprising mature erythrocytes, PCEs and the MN-PCEs (Fig. 7B). TBI caused a significant decline in the frequency of PCEs at 24 hours (0.46% ± 0.15; p < 0.01), 48 hours (0.27% ± 0.033; p < 0.001) and 72 hours (0.29% ± 0.019; p < 0.0001) in comparison with the naive mice at 24 hours (5.953% ± 1.007), 48 hours (4.725% ± 0.38) and 72 hours (4.575% ± 0.315) respectively (Fig. 7C). DAMTC enhanced the frequency of PCEs by 2.5 to 6 folds at all the evaluated time points (1.18% ± 0.19; p < 0.05 at 24 hours, 1.95% ± 0.14; p < 0.001 at 48 hours and 1.57% ± 0.233; p < 0.01) as compared to the values in TBI mice, indicative of reduced cytotoxicity. In accordance with DAMTC-mediated reduction in TBI-induced cytotoxicity, there was a concomitant alleviation of radiation-induced genotoxicity as visualised by diminution in the frequency of MN-PCEs with DAMTC treatment (Fig. 7C). At all the aforementioned time points, DAMTC significantly reduced the radiation-induced MN frequency by 6 folds at 24 hours, 4 folds at 48 hours and 2 folds at 72 hours (Fig. 7C). These results strongly suggest the ability of DAMTC to ameliorate the radiation-induced cytotoxic and genotoxic insults to the hematopoietic system, thereby mitigating TBI-induced lethality in C57BL/6 mice.

#### Reduction in apoptotic cell death and enhancement of cell proliferation in the bone marrow

**Figure 8:**
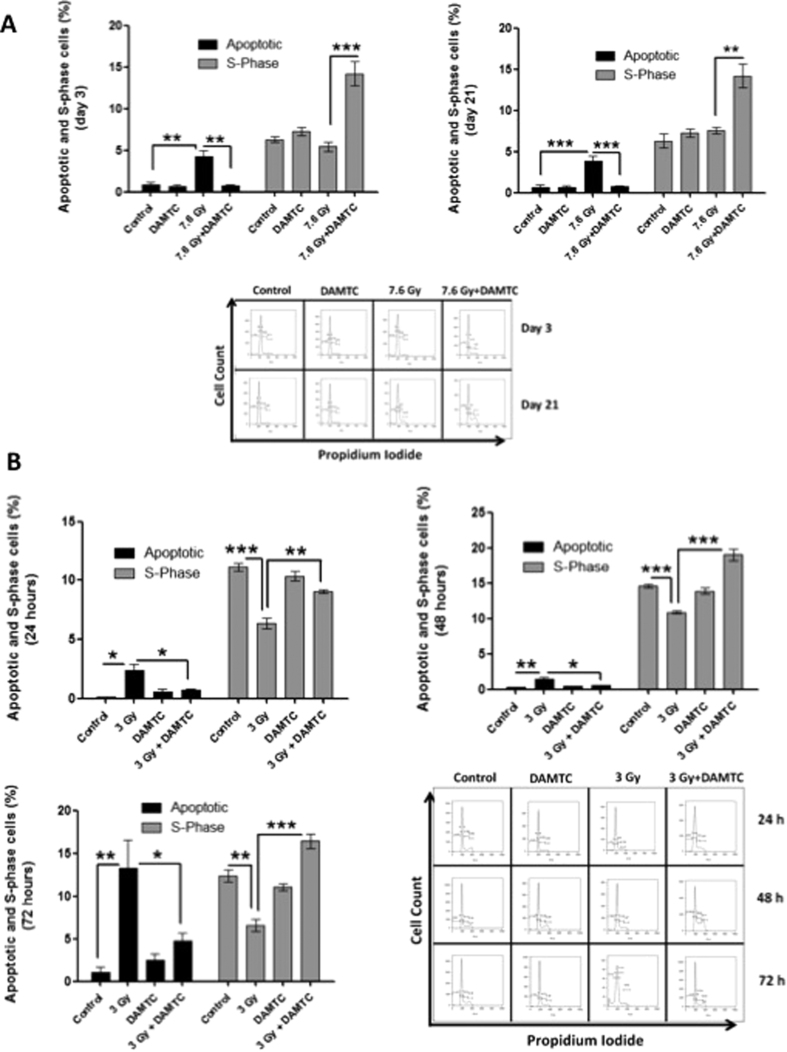
Reduction in the radiation-induced apoptosis and stimulation of cell proliferation in the BM by DAMTC. Effects of DAMTC on TBI-mediated apoptotic death and compromised proliferative ability in BM was evaluated by analysis of hypo-diploid cells and S-phase cells respectively in the BM. (**A**) Frequency of apoptotic and S-phase cells at days 3 and 21 post TBI (7.6 Gy) in mice (n = 10 in all groups), along with representative DNA flow cytograms. (**B**) Average values of apoptotic and S-phase cells and representative DNA flow cytograms observed at 24, 48 and 72 hours following treatment in naïve, TBI (3 Gy) and TBI + DAMTC mice (n = 10 in all groups). All error bars indicate SEM. *P < 0.05; **P < 0.01; ***P < 0.001.

Apoptosis, cell proliferation and differentiation are key processes regulating size of hematopoietic lineages and maintaining the homeostasis in the hematopoietic system^6^. Since apoptosis of BM cells is involved in the onset of H-ARS, while entry of stem cells into the cell cycle results in hematopoietic recovery, we analysed the effects of DAMTC on TBI-induced apoptosis and proliferation in BM by studying the cell cycle progression. At day 3, there was a notable increase in the percentage apoptotic cells in the marrow from TBI mice, (p < 0.01) which was significantly reduced (p < 0.01) in DAMTC-treated irradiated mice. DAMTC treatment significantly enhanced the cell proliferation in the irradiated animals (p < 0.001) at day 3 post TBI (Fig. 8A). DAMTC treatment post TBI caused profound reduction in apoptotic cell death (p < 0.001) in comparison with the TBI mice as observed on day 21. Interestingly, a concomitant drastic enhancement in proliferation by DAMTC was also visualised (p < 0.05) when compared with the TBI cohort (Fig. 8A). These results suggested that even at day 21, the TBI mice were incapable of combating the TBI-induced damage (higher apoptosis and lower cell proliferation) as opposed to the mice administered with DAMTC. At an absorbed dose of 3 Gy, a significant increase in the percentage apoptotic cells was observed (p < 0.05 at 24 hours; p < 0.01 at 48 and 72 hours), which was reduced by nearly 4 folds in animals that received DAMTC (p < 0.05) (Fig. 8B). Under these conditions, DAMTC also caused a significant up-regulation in the cell proliferation in the irradiated animals (p < 0.01 at 24 hours, p < 0.01 at 48 hours and p < 0.001 at 72 hours) (Fig. 8B). Cumulatively, these results suggest that DAMTC up-regulates proliferation and down-regulates apoptotic cell death in the BM which appear to serve as potential contributing factors for the mitigation of TBI-induced hematopoietic injury (H-ARS).

#### Induction of M1 macrophages in the spleen and stimulation of immuno-protection

**Figure 9:**
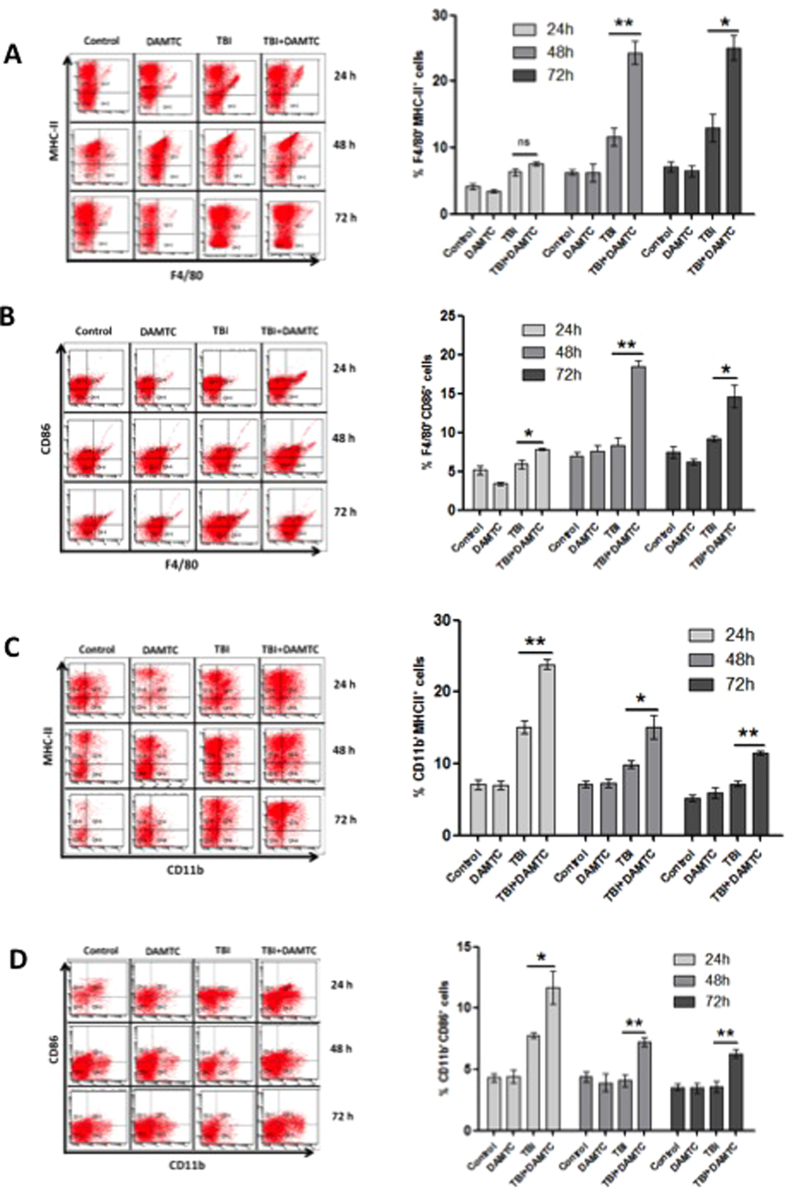
DAMTC-facilitates the induction of M1 macrophages in the spleen of TBI mice. Splenocytes were isolated at 24, 48 and 72 hours following treatment and stained for macrophagic markers (F4/80 and CD11b) and antigen-presenting/co-stimulatory markers (MHC-II and CD86). Results were analysed as double-positive cells. Representation of frequency of (**A**) F4/80^+^MHC-II^+^ cells (**B**) F4/80^+^CD86^+^ cells (**C**) CD11b^+^MHC-II^+^ cells and (**D**) CD11b^+^CD86^+^ cells (n = 10 for naïve, DAMTC, TBI (3 Gy) and TBI + DAMTC groups). All error bars indicate SEM. *P < 0.05; **P < 0.01; ***P < 0.001.

Macrophages are indispensable and crucial element of the innate immune system that orchestrate initial responses to any kind of damage-causing stimulus, and additionally the local inflammatory process mounted to counteract the radiation-induced damage is characterized by the involvement of the macrophages in a plethora of functions such as antigen presentation, phagocytosis, cytokine secretion etc.^61^,^62^. Accumulation of monocytes and their differentiation into mature macrophages (M1) is one of the hallmarks of the effector phase of inflammation^63^,^64^. To determine the effect of DAMTC on the macrophages in response to TBI, we studied the expression of macrophage cell surface markers (F4/80 and CD11b) and antigen-presenting/co-stimulatory molecules (MHC-II, CD86) in mouse splenocytes (Fig. 9). An upregulation of F4/80^+^MHCII^+^cells was evident in mice that received DAMTC following irradiation compared with the TBI mice, although it was significant at 48 hours (p < 0.01) and 72 hours (p < 0.05) (Fig. 9A). In accordance with the upregulation of F4/80^+^MHCII^+^cells, a concomitant accretion of F4/80^+^CD86^+^ cells that participate in the primary phase of immune response was visualised at all three post-treatment times with the maximum level observed at 48 hours (p < 0.01) (Fig. 9B). Furthermore, DAMTC treatment also resulted in augmentation of the CD11b^+^MHCII^+^ macrophages (p < 0.01 at 24 and 72 hours; p < 0.05 at 48 hours) (Fig. 9C). A simultaneous upsurge of CD11b^+^CD86^+^ (p < 0.05 at 24 hours; p < 0.01 at 48 and 72 hours) macrophages was also noted in animals that received DAMTC following TBI as compared to their TBI counterparts (Fig. 9D). These observations underscore the potential of DAMTC in enabling differentiation and activation of M1 macrophages to offset H-ARS following TBI.

## Discussion

Management of radiation injuries is a complex medical challenge, requiring careful use of mitigation as well as therapeutic agents, administered at appropriate times following the exposure. Orchestrated proliferation and differentiation of surviving stem cells and re-population by the hematopoietic progenitors (of different lineages) following TBI are the two main factors responsible for the reconstitution of the hematopoietic system, thereby restoring the immune competence, facilitating animal survival. Currently, available evidences support the notion that various colony stimulating factors viz. G-CSF, GM-CSF etc. significantly facilitate recovery from radiation-induced hematopoietic injury and thus, enhance the survival of individuals experiencing ARS^12^. Results of the present study, clearly demonstrate the ability of DAMTC to stimulate the splenic (Fig. 3) progenitors, as well as the myeloid progenitors in the BM like CFU-GM, CFU-GEMM and BFU-E (Fig. 5) facilitating hematopoietic repopulation, thereby, supporting the proposition that DAMTC is highly competent in ameliorating the radiation-induced hematopoietic injury by virtue of facilitation of recovery. Its ability to stimulate proliferation (Figs 4 and 8) in the BM, as well as differentiation of the M1 macrophages in the spleen (Fig. 9), further strengthens this proposition. Moreover, the ability of DAMTC to expedite recovery of the hematopoietic system seen here suggests that it is able to stimulate the proliferation and differentiation of spared stem cells in a regulated manner thereby, repopulating the blood and the BM through the hematopoiesis, mitigating TBI-induced lethality in mice.

The remarkable ability of DAMTC to mitigate acute radiation effects when administered (i.p.) 24 hours following lethal TBI (Fig. 1) makes it an attractive radiation-countermeasure agent for human use in the event of an accidental radiation exposure as well as in certain instances of planned exposures. The dose-modifying factor (DMF) for mitigation observed in the current study (1.382 at LD_50/30_) in C57BL/6 mice (Fig. 1) indeed merits consideration for its further development as a potential countermeasure agent for mitigation of radiation injury.

Polyphenols are known to be cyto-protective, by virtue of their anti-oxidant properties at low concentrations/dose, while they function as pro-oxidants at higher levels^40^. At higher concentrations, polyphenolic acetates (like DAMC and DAMTC) also show anti-tumor and radio-sensitizing effects in tumor cells^40^,^47^. In the present study, we deployed the lowest possible dose of DAMTC (5 μg/kg), which elicited a moderate protective effect (50–60% mice survival), but a substantial degree of mitigation (80% mice survival) against TBI (Fig. 1), which correlated well with the recovery from hematopoietic injury. It would however, be worthwhile screening a range of doses and exploring the dose-dependent effects of DAMTC against TBI.

The hematopoietic (H-ARS) and the gastro-intestinal (GI-ARS) sub-syndromes constitute the clinical components of ARS^6^,^12^. The H-ARS is characterized by severe cytopenia and suppression of the hematopoietic compartments^12^,^17^. The GI-ARS following TBI (>6 Gy), is characterized by massive intestinal ablation due to apoptosis and its pathophysiological manifestations include mucosal barrier disintegration, vascular damage, enterocyte depletion resulting in increased susceptibility to bacterial infections causing sepsis, immune-suppression and eventually death^12^. Increasing evidences suggest that TBI (<10 Gy) causes prolonged and profound pancytopenia ensuing in mortality of mice following haemorrhage or chronic opportunistic infections^10^,^11^. Consistent with previous findings, we observed decrease in almost all hematological parameters in the blood at days 3, 7, 14, 21 and 28 after TBI of 7.6 Gy, which was the LD_50/30_. DAMTC moderately increased the RBC and platelet counts coupled with a major recovery in hemoglobin attaining baseline levels 21 days following TBI. Strikingly, significant accretion in WBC (12 folds) and lymphocyte (>2folds) numbers at day 21, and sustained levels at day 28 (Fig. 2), on DAMTC treatment indicated accelerated hematopoietic recovery, lowered vulnerability to opportunistic pathogens, infections as well as enhanced immune-competence accounting for reduced lethality and improved survival at lethal TBI. It is intriguing that recovery was not visualised at earlier time points assessed in the study, until day 21, and this may be attributed to the mitigation regimen (DAMTC treatment 24 hours after TBI). Hence, the time-lapse between significant damage caused viz. plummeting blood cell indices and drug administration may account for the time taken in restoration to baseline levels after IR-induced hematopoietic injury.

IR causes debilitating impairment of every hematopoietic compartment^12^,^17^ that can be revealed by endogenous CFU assay. A significant increase in CFUs (~4 folds), and absence of splenic atrophy, at day 10 post TBI (7.6 Gy) with DAMTC treatment (Fig. 3), provided clue not only for mitigation of TBI-induced damage to the splenic compartment but also proficiency of DAMTC in preserving the stemness of the splenic progenitor cells. Accumulating evidences suggest onset of a hematologic crisis, ensuing soon after exposure to TBI, resulting in BM hypoplasia or aplasia^13^,^56^ manifesting eventually in BM failure, a hallmark of H-ARS^6^,^12^. The potential of DAMTC to completely offset the TBI (7.6 Gy)-induced loss of cellularity in the BM (day 10), replenishing the cellular content and restoring the cellular integrity of the BM, which was corroborated with a 2-fold increase in the BMNC count at day 10 (Fig. 4), provided evidence for DAMTC-induced hematopoiesis, thereby, facilitating stem cell regeneration in the BM. This may be one of the several plausible contributions for the mitigation of H-ARS and TBI-induced lethality by DAMTC. Hematopoietic toxicity manifesting as BM-failure following irradiation occurs largely owing to severe destruction of the hematopoietic progenitors, such that, for amelioration of H-ARS, marrow reconstitution of BM-hematopoietic progenitor cells (HPCs) is not only indispensable, but a pre-requisite for BM recovery post myelo-ablative TBI^6^,^10^,^12^. DAMTC-mediated significant increases in CFCs from different myeloid progenitors (CFU-GM, CFU-GEMM, BFU-E) reveal expedition of multi-lineage recovery to alleviate TBI-induced partial or complete diminution of CFCs (Fig. 5). This is also indicative of the stimulation of BM hematopoiesis by enabling preservation and/or induction of HPCs and protection of the hematopoietic stem cell compartment to mitigate H-ARS by virtue of reversal of BM-suppression.

Apoptosis, cell proliferation and differentiation, are key processes that work in a cohesive manner to regulate the population of hematopoietic lineages and further maintain hematopoietic homeostasis^6^. Increasing number of evidences suggest that one of the primary causes for H-ARS following TBI is induction of apoptosis in the BM cells^6^,^10^,^12^. Reduction in the apoptotic cell death in the BM following sub-lethal TBI (7.6 Gy and 3 Gy; Fig. 8) accompanied by an upsurge in cell proliferation (Fig. 8) suggest DAMTC-mediated facilitation of entry into the cell cycle of self-renewing and differentiating HSCs in the BM, to safeguard the various hematopoietic compartments, as a part of the mitigation against H-ARS.

While radiation-induced interphase death occurring by apoptosis contributes to the TBI-induced cell death in different organs, mitotic death contributes majorly to TBI-induced lethality, particularly at low and moderate doses^2^. IR-induced DNA damage, resulting in chromosomal aberrations (unrepaired, mis-repaired or incompletely repaired double-stranded breaks) manifests as micronuclei in the daughter cells and such cells are mostly associated with IR-induced mitotic cell death^40^,^60^,^65^. The *in vivo* MN assay serves as a reliable measure, and most widely accepted model for IR-induced cytogenetic damage (chromosome loss and breakage) and genotoxicity^65^,^66^,^67^. IR-induced micronuclei, in the polychromatic erythrocytes (MN-PCE), in the peripheral blood and BM, are an indicative measure of induced chromosomal damage in cells of the hematopoietic system^57^,^58^,^68^. Decrease in the frequency of MN-PCEs in the BM (Fig. 6) and blood (Fig. 7) with concomitant increase in the frequency of PCEs in blood (Fig. 7) suggests the ability of DAMTC to mitigate IR-induced genotoxicity and cytotoxicity respectively, thereby reducing the mitotic death in different hematopoietic compartments. However, it is to be noted that reduction in the micronuclei fraction seen in the polychromatic erythrocytes is not sufficient for concluding the effects of DAMTC on the radiation-induced cytogenetic damage in other cell populations of the BM and will require further studies to unravel this potential. Nevertheless, reduction in the mitotic death by DAMTC, also appears to play a role in the mitigation of hematopoietic injury following radiation exposure. Radiation-induced micronuclei formation is suggestive of mitotic catastrophe and reflects genomic instability, which also contributes to the late effects in survivors. Reduction in the radiation-induced cytogenetic damage in the marrow cells and blood suggests that DAMTC can reduce the late effects on the hematopoietic system as well, besides mitigating the hematopoietic acute radiation effects shown here. Further studies are required to establish this potential of DAMTC.

Hematologic crisis, characteristic of H-ARS has deleterious consequences on proper functioning of the immune system thereby, increasing vulnerability to opportunistic infections and is associated with the observed lethality following TBI^1^,^10^. Macrophages are crucial players of the innate immunity that orchestrate initial responses to any kind of damage causing stimulus^64^. Moreover, it is essentially the recruitment of monocytes and their differentiation into mature macrophages (M1 phenotype) that triggers the effector phase of immune response particularly the induction of inflammation to combat the injury^63^,^64^. The effector phase is in turn mediated by phagocytosis (of microbes, antigens, immunogenic molecules), cytokine secretion and priming other immune cells by providing processed antigens and signals to stimulate their maturation and functions^61^,^62^. Up-regulation of F4/80^+^MHC-II^+^ and F4/80^+^CD86^+^ macrophages in splenocytes brought about by DAMTC following TBI (at 24–72 hours) coupled with an upsurge in CD11b^+^MHC-II^+^ and CD11b^+^CD86^+^ macrophages (Fig. 9), are clearly suggestive of the potential of DAMTC in enabling differentiation and activation of M1 macrophages thereby, exhibiting immune-stimulatory properties to offset detrimental immuno-compromised condition following TBI-induced hematopoietic injury. The enhanced expression of specific macrophage subsets seen here can facilitate antigen presentation and cytokine secretion favouring activation of a cascade of cellular events to confer immune-protection. It appears, therefore, that DAMTC mitigates lethality and immuno-toxicity associated with H-ARS by induction of specific macrophagic subsets. Further studies, however, are warranted to elucidate the cascade of events following macrophage activation by DAMTC to understand the detailed mechanism of DAMTC-mediated immuno-stimulation and immuno-modulation post TBI.

Enhanced antioxidant defence^40^,^50^, alterations in the protein (histone) acetylation^42^,^43^,^44^,^47^ leading to changes in the epigenetic regulation and facilitation of DNA repair^46^ are among the possible mechanisms that could contribute to the amelioration of radiation-induced injury to the hematopoietic system and enhanced survival observed here. Future studies should focus on the understanding of the mechanisms underlying mitigation of radiation induced injury to the hematopoietic system, so as to optimize the use of DAMTC or any other PA, which may have the potential to modify redox status as well as signalling processes related to damage response in cells. DAMTC is a redox molecule, and it would be interesting to examine its ROS-scavenging abilities in the context of the present study to account for the ability to mitigate TBI-induced hematopoietic injury. Accumulating evidences suggest that upregulation of ROS is one of the leading causes for HSC senescence^6^,^69^,^70^,^71^ as observed under different pathological conditions. Recent studies have shown that aging-dependent hematopoietic failure exhibited in ATM^−/−^ mice was reversed by N-acetyl-cysteine (ROS scavenger) which restored HSC function and prevented BM failure condition^69^. The importance of scavenging/quenching delayed ROS in the context of mitigating IR-induced injury, and H-ARS is immense, and requires studies examining the temporal changes following radiation exposure. It would be worthwhile, to investigate the same with DAMTC, and also monitor the pharmacokinetics to establish the relationship between its circulating levels and mitigation of damage to facilitate studies in non-human primates required under the “two animal rule” of the FDA and safety in humans, before it can be considered suitable for mitigation of radiation damage in human subjects.

Multiple organ failure (MOF), as a consequence of uncontrolled inflammatory responses coupled with the damage to the vascular system contributes to the acute effects of TBI, and constitutes an important aspect of radiation injury^72^. While, injury to hematopoietic system does compromise the immune system, pre-disposing the exposed individuals to infection, amelioration of H-ARS when combined with MCM agents that can reduce damage to GI and other organs as well (thereby reducing the degree of multiple organ dysfunctions) would be very effective for the mitigation of radiation injury. Indeed, our preliminary observations revealed that DAMTC mitigates the TBI-induced GI damage (data not shown). Furthermore, polyphenols and other polyphenolic acetates like 7, 8-diacetoxy-4-methylcoumarin (DAMC) have been shown to stimulate angiogenesis through activation of nitric oxide synthase and immune system^73^. Therefore, DAMTC appears to have the potential for reducing radiation-induced MOF and hence, be developed as an MCM to mitigate ARS. It also has a potential to be developed as an adjuvant to radiotherapy for protecting the normal tissues and thus enhance the therapeutic gain. Further studies are required to investigate DAMTC induced temporal changes in the inflammation following radiation exposure, before contemplating DAMTC as a mitigation agent in case of accidental human exposure to radiation. Likewise, its ability to offset damage to the normal tissues during focal irradiation of tumors in the fractionated regimen needs to be evaluated for establishing it as an adjuvant to radiotherapy.

In summary, the results of the present studies, clearly demonstrate that DAMTC when administered 24 hours post TBI effectively mitigates radiation-induced hematopoietic injury, myelotoxicity and BM suppression, which is contributed by multiple factors viz. (i) accelerated recovery of lymphocytes and WBCs, (ii) augmentation of the numbers and functions of the hematopoietic progenitors in BM and spleen, (iii) restoration of BM cellularity by decreasing apoptotic cell death and stimulating proliferation (iv) reduction in IR-induced cytogenetic damage in BM and blood, and (v) most importantly enhanced immuno-protection by up-regulating expression of M1 macrophages in the spleen. These observations unequivocally establish the potential of DAMTC as a countermeasure agent to mitigate radiation-induced hematopoietic injury and consequently, TBI-induced lethality. Further studies are warranted to unravel the mechanisms underlying reduction in the radiation-induced apoptosis and cytogenetic damage linked to the repair of DNA damage (strand breaks) by DAMTC, besides its ability to provide immune protection.

## Additional Information

**How to cite this article**: Venkateswaran, K. *et al.* Mitigation of radiation-induced hematopoietic injury by the polyphenolic acetate 7, 8-diacetoxy-4-methylthiocoumarin in mice. *Sci. Rep.* **6**, 37305; doi: 10.1038/srep37305 (2016).

**Publisher’s note**: Springer Nature remains neutral with regard to jurisdictional claims in published maps and institutional affiliations.
