# Author Correction: Mitigation of radiation-induced hematopoietic injury by the polyphenolic acetate 7, 8-diacetoxy-4-methylthiocoumarin in mice

**DOI:** 10.1038/s41598-021-02777-z

**Published:** 2021-11-30

**Authors:** Kavya Venkateswaran, Anju Shrivastava, Paban K. Agrawala, Ashok Prasad, Namita Kalra, Parvat R. Pandey, Kailash Manda, Hanumantharao G. Raj, Virinder S. Parmar, Bilikere S. Dwarakanath

**Affiliations:** 1 Division of Metabolic Cell Signalling Research, Institute of Nuclear Medicine and Allied Sciences, Brig. S. K. Mazumdar Marg, Lucknow Road, Delhi, 110054 India; 2 Department of Zoology, University of Delhi, Delhi, 110007 India; 3 Department of Chemistry, Bioorganic Laboratory, University of Delhi, Delhi, 110007 India; 4 Department of Biochemistry, VP Chest Institute, University of Delhi, Delhi, 110007 India; 5 Central Research Facility, Sri Ramachandra University, Porur, Chennai, 600116 India

Correction to: *Scientific Reports* 10.1038/srep37305, published online 16 November 2016

The original version of this Article contains an error in Figure 5, where the right image in the Control group is a duplication of the middle image in the 7.6 Gy+ DAMTC in panel A. The correct Figure 5 and accompanying legend appear below.

**Figure 5:**
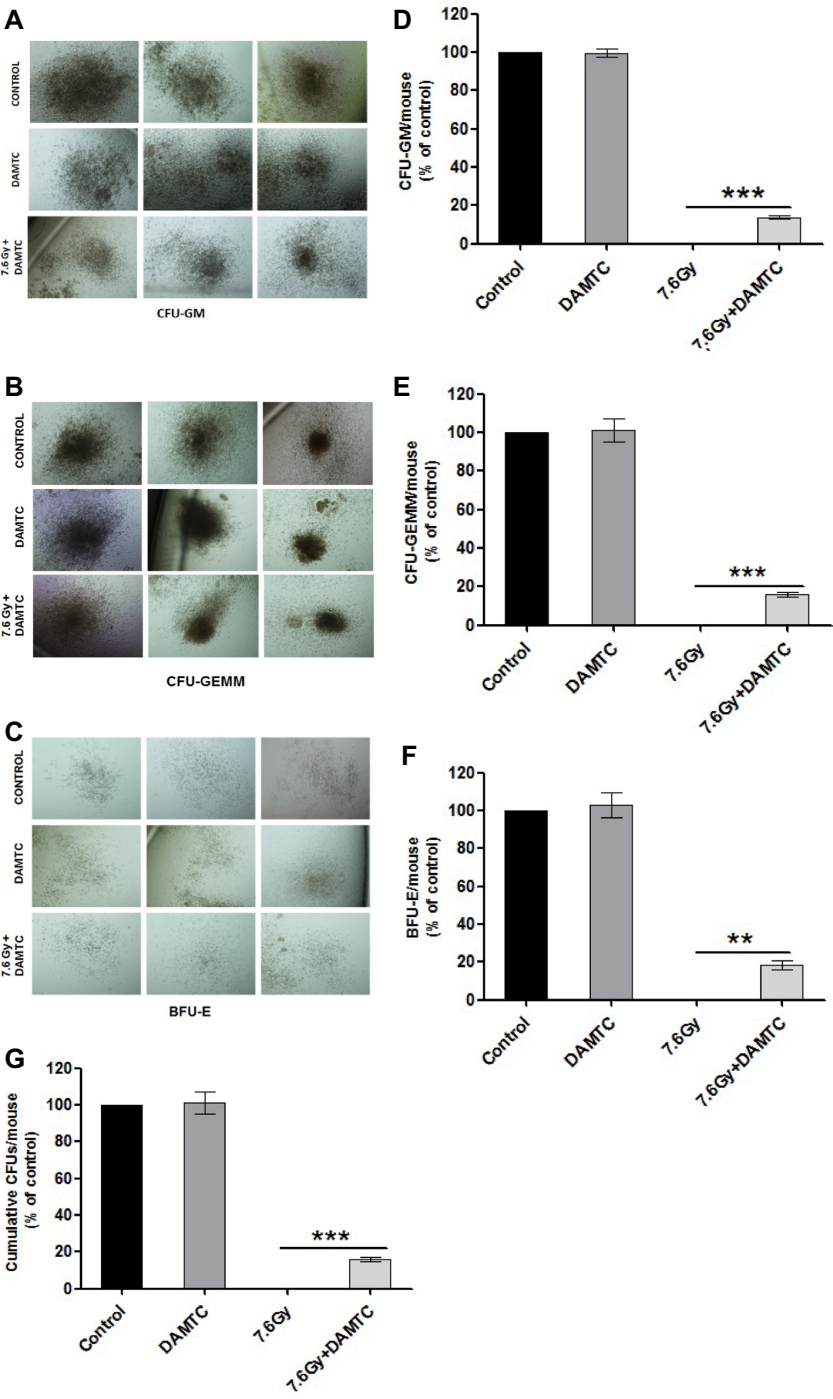
DAMTC facilitates expansion of hematopoietic progenitors in the BM of TBI mice. Effects of DAMTC on BM hematopoietic progenitor cells (HPCs) in TBI mice. Panels show colonies of hematopoietic progenitors (**A**) CFU-GM, (**B**) CFU-GEMM and (**C**) BFU-E after performing *ex-vivo* culturing on day 10 post TBI. Representative images of colonies from naïve, DAMTC and TBI + DAMTC mice are shown (cells from ten animals were examined in each group; n = 10). Percentages of (**D**) CFU-GM, (**E**) CFU-GEMM, (**F**) BFU-E and (**G**) cumulative CFUs are shown. All error bars indicate SEM. *P < 0.05; **P < 0.01; ***P < 0.001. Imaging of BM hematopoietic CFUs was done on day 12 of *ex-vivo* culture. Original magnification, ×40 (**A**–**C**).

